# Book Review

**Published:** 1930

**Authors:** 


### Reviews of Books

**Through the States with a Seeing Eye.** By R. J. A. Berry, M.D., F.R.C.S., F.R.S. Pp. 200. Illustrated. Bristol: John Wright & Sons Ltd. 1930. Price 5s.—This is a most entertaining little book and makes one long to know the author, a longing which every Bristol man can now gratify as Dr. Berry has taken up his residence among us and the appointment of Medical Superintendent of the Stoke Park Colony. The author was one of a deputation sent from Australia to America to investigate matters relating to hospitals, medical education and universities. On his return to Australia he was asked to broadcast his impressions of the States. This he did in twenty-five talks which constitute the present volume. He is bright and breezy and yet most informative. His banter is always good-natured. Unfortunately for medical readers, the technical aspects of the problems he was sent to study are here omitted, and we are merely treated to the spectacle of a grey-haired school-boy having a jolly good holiday; and we only wish that he would personally conduct us over this or any other foreign tour.

**The Sthenics.** By Sir James K. Fowler, K.C.V.O., C.M.G., M.D. Pp. 81. London: Macmillan & Co. Ltd. 1930. 3s. 6d.—The tides of practice retired, Sir James K. Fowler ruminates on man in life's fitful fever; delineates a type, and assigns its physiology. "Sthenics" is not the echo of "neurasthenics." The sthenics are men of "sthenical affections" (as Kant has it), and have the marks of vitality, exuberance, and versatility, are keen of sense to a fault, and egotistical. Curious instances of hypersensitiveness are given, and a gastric crisis described, of adrenal origin, the infirmity, it is argued, mistaken in Napoleon for epilepsy. A lay public loves the anatomy of genius and the play of a hidden secretion upon career and character. Sir James Fowler's book is too brief for so full a fancy; he has admired the scientific vision of Dr. Budd of Bristol (by the way Dr. Budd is described as "attached to the General Hospital"; actually he was Physician to the Royal Infirmary 1847–62), felt in the fields of France the spell of Jeanne d'Arc, and seen the reins of power in the hands of Curzon and Haldane.

**Applied Physiology.** By SAMSON WRIGHT, M.D., M.R.C.P. Third edition. Pp. xxviii., 552. Illustrated. London: Oxford University Press. 1929. Price 18s.—The appearance of a fresh edition of this work twelve months after the last is sufficient testimony to its popularity. In this, the third edition, the whole has been revised, much additional material being added and some of the sections considerably altered. A very valuable appendix on the conditioned reflexes has been included; this is an able synopsis of Pavlov's monumental work. The inclusion of such wealth of material has resulted in some of the sections, more particularly perhaps those dealing with the nervous system and with the body chemistry, being so elaborate and detailed that it is impossible not to feel that the book now rather tends to defeat its own ends. Controversial and minor points, which at the moment, at any rate, can only have an academic interest, are presented in such detail that the work has become rather a valuable book of reference for the pure physiologist than a concise statement of the essential facts of physiology as they concern the clinician. Some of the tables copied from other authors appear to have suffered in the transfer. For example, the table describing the chemistry of physiological and pathological cerebro-spinal fluids, which is said to be after Symonds, shows small but very important variations from the tables published by C. P. Symonds in the *Clinical Journal.*

**Le Sinus Carotidien et les autres zones Vasosensibles Réflexogènes.** By Dr. CORNEILLE HEYMANS. Pp. 121. Illustrated. London: H. K. Lewis & Co. Ltd. 1929. Price 5s.—In this interesting monograph is described a series of ingenious experiments proving that the variations in heart rate and systemic blood pressure, which follow alterations in the cephalic blood pressure, are due, not to direct stimulation of the cardiac and vasomotor centres, but to a reflex, the receptor organ of which is the carotid sinus and carotid body. The experiments would appear to prove this thesis conclusively. However, the author invokes the suprarenals as playing a part in the vasomotor mechanism of this reflex, and holds the view that a continual secretion of adrenalin is responsible, in part at all events, for the maintenance of normal vasomotor tone. This is not in accord with the current English views, and his conclusions on this point cannot be accepted too freely. There is a full bibliography of the anatomy and physiology of the carotid sinus and carotid body. These researches should be of great interest not only to physiologists but also to pathologists and clinicians ; but before the thesis can be accepted as proved the work obviously requires confirmation from other workers.

**Introduction to the study of the Nervous System.** By E. E. HEWER, D.Sc., and G. M. SANDES, M.B., B.S. Pp. xii., 104. 53 illustrations. London : Wm. Heinemann Ltd. 1929. Price 21s. Not only all the innate reflexes of man, but also those which have been termed "conditional," for which the integrity of the cerebral hemispheres is essential, are necessarily dependent upon a definite architecture of the nervous system. In this volume the text and diagrams are the outcome of long teaching experience. Although there is a distinct danger that the student may store such diagrams in his mind to the exclusion of the actual arrangement of the systems as these exist in the body, it is still true that only by diagrams can such complicated connections as those of the cerebellum for example be made clear. A careful search has failed to reveal misstatements in the text or errors in the figures, praise that few text-books deserve. The authors have succeeded in producing a really valuable book and one that can be recommended with confidence to students, teachers, and practitioners alike.

**Acute Infectious Diseases.** By J. D. ROLLESTON, M.D., M.R.C.P. Second Edition. Pp. xi., 419. London : Wm. Heinemann Ltd. 1929. Price 15s.—The first edition of this work appeared in 1925, and was based on twenty-five years' experience as a medical officer under the M.A.B. This year Dr. Rolleston has brought out a second edition, and has embodied all the recent advances made during the last four and a half years. He writes critically and informatively on both the Schick and Dick re-actions in the diagnosis of diphtheria and scarlet fever respectively. From his own experience of the two tests he regards the Schick test as far the more reliable. He does not consider the practitioner is under the same obligation to recommend active immunisation against diphtheria as he is to urge vaccination against small pox. Attention is drawn in the chapter on vaccination to the reported cases of nervous sequels which have followed vaccination, more especially in this country and Holland. Attention is also called to the recommendations of the Committee, appointed by the Ministry of Health, with the hope of lessening these disasters. Every chapter of the book is well written and full of well-digested knowledge, which is invaluable to every practitioner in carrying out his daily task.

**Testicular Grafting from Ape to Man.** By SERGE VORONOFF. Pp. viii., 125. Illustrated. London: Brentano. 1929.—This small book gives a full account of the author's method of rejuvenating old men by grafting ape's testicle on to the surface of the human tunica vaginalis. The histological evidence is given that such grafts survive, at any rate for about four years. The ingrafted testis is cut into four, like splitting up the sections of an orange, and two pieces implanted on the rawed surface of each tunica vaginalis. Only a minute or two should elapse between dividing the vascular attachments of the animal gland and sewing it on to the human. It is claimed that as a result of 475 cases treated since 1920, success was usually attained lasting about five years; a brief psychical improvement during the first few days not being counted. The results are given as follows: 236 cases of premature senility, 10 per cent. failed, 90 per cent. success; 64 cases of senility, 26 per cent. failed, 74 per cent. success; 28 cases of impotence, 11 per cent. failed, 89 per cent. success; 14 cases of impotence due to organic causes, 43 per cent. failed, 57 per cent. success. Opportunities are scarce in this country to obtain suitable material for the graft, as the ape must be neither too young nor too old; we think, however, that we must allow that the writer makes out a sufficiently good case to warrant a trial of his method when it is possible.

**Hæmodynamics.** By P. B. KITTEL, F.R.C.S. Pp. xi., 195. Illustrated. London: H. K. Lewis & Co. Ltd. 1929. Price 10s.—The above title covers the physiology and treatment of varicose veins. After describing the three types of varicosity, the author discusses the action of the valves. He holds that incompetence of the valves results from, and is not the cause of, dilated veins. He states that if œdema, following venous thrombosis, persists for a year, it will probably be permanent. He believes that varicose ulcers arise from the hydrostatic effect of the œdema upon the nerves, the lesion being extended later by secondary infection. His chapters on treatment are based on 446 cases covering 2,731 injections into the veins. No tourniquet is used. The skin is painted with iodine. The patient is recumbent for treating large veins, sits down for smaller veins, and stands for the smallest ones. Injections are started at the calf level, avoiding vessels over the tibia. The quantity of solution used depends on the drug. The patient then lies down for a minute and a light bandage with wool pad may be applied. With regard to the drug, the author has tried many. He discarded salicylic acid and quinine as they were liable to be painful and sometimes produced ulcers. Borocaine and glucose were found to be ineffectual but harmless. He then turned to sodium morrhuate in 5 and 10 per cent. watery solutions. With this he finds the veins become blocked and hardened in a few minutes, and ulcers do not follow. About eight injections are usually required. This book differs from the extensive literature which has appeared on this subject by the detailed discussion on the mechanism of venous, capillary and lymphatic flow and its relation to œdema.

**Varicose Veins.** By T. H. Treves-Barber, M.A. Pp. 120. 11 illustrations. Bristol: John Wright & Sons Ltd. 1929. Price 6s.—Dr. Barber in this little book, based chiefly on personal observation and practice, sets out to describe to the uninitiated the injection treatment of varicose veins. We feel that those who follow carefully his teaching and take heed of his warnings should have no difficulty in putting this method of treatment into practice. As the author states, there is as yet no ideal sclerosing solution. He advocates from personal experience a hypertonic saline solution in preference to the more generally used quinine and urethrane solution and apparently has found no cause to regret his choice. We can recommend this book to the general practitioner.

**Orthopedic Surgery.** By Sir Robert Jones, Bart., K.B.E., C.B., Ch.M., F.R.C.S., and R. W. Lovett, M.D., F.A.C.S. Second edition. Pp. 807. 792 illustrations. London: Oxford University Press. 1929. Price 52s. 6d.—This standard book, representing the mature views of the two greatest authorities of orthopedic surgery in England and America, was first published five years ago, and represents one of the happy results of that close co-operation between the chief countries of the English-speaking world which took place during the war. It was based upon the wider definition of orthopedic surgery agreed to at that time, and dealt therefore not merely with deformities, but with surgery of bones and joints, muscles and nerves. The present edition has lost, by death, the guiding hand of the distinguished American author, whose place has been taken by two well-known orthopedists from Harvard University, whilst the younger generation in this country has been represented by Mr. Harry Platt of Manchester. The following represent the chief additions to the new edition. In Chapter v, on stiffness and ankylosis of the joints, a Putti's operation for arthroplasty of the knee is described and illustrated, but to this description is added a word of warning as to dangers and limitations. In Chapter xi the operative treatment of arthritis deformans is given a greater prominence, special reconstructive operations on the hip being described. In Chapter xvi the developmental diseases of bones is more fully dealt with, and it is to be remarked that rickets is included in this heading. In Chapter xvii on affections of adult bones, the recent work of the American collective investigation of bone sarcoma is included. In Chapter xxi on poliomyelitis, additions, chiefly descriptive of operations for the substitution of paralysed muscles, have been made. We note with some surprise that the operation for substituting tensor fasciae femoris for the paralysed glutei described by Hey Groves in his Bradshaw Lecture of 1926 is attributed to Ober, whose article appeared in April, 1927. In Chapter xxxii on scoliosis, many of the modern methods of forcible correction of the deformity, including that of Galeazzi, are described, but these are accompanied by a wise warning as to length of time needed for exercises and after-treatment in order to make such methods worth considering. The many clear illustrations greatly add to the value of the book, and of these some of the best are those X-ray pictures provided by Mr. Thurstan Holland. The great and outstanding value of the work is that it gives the considered judgment of two of the wisest orthopedic surgeons. That this judgment, if it ever errs, does so in the direction of conservatism is perhaps an advantage. The defect of the book is that it does not give a sufficiently full account of modern operative methods, *e.g.* those of bone grafting, or those used for ununited or malunited fractures.

**Recent Work on Colporrhaphy, Rheumatism and Coli Bacilluria.** By E. H. Roberts, F.R.C.S. Pp. 28. Illustrated. London: H. K. Lewis & Co. Ltd. 1929. Price 3s.—This is an interesting monograph divided into four parts. Part I. is a description of the author's technique for the operations of anterior and posterior colporrhaphy ; it is clear and well illustrated, although possibly overshadowed by Ward's recent work in Kelly's *Gynæcology*. Part II. is a follow-up of fifty-two cases, showing good results, and also introducing the relation of rheumatism to cystocele. In Parts III. and IV. the author elaborates this relationship, dwelling on the causal possibilities of coli bacilluria in rheumatism, but rather surprisingly seems to suggest that a cystocele is an effect and not a cause of chronic rheumatism.

**The Hygiene of Marriage.** By I. E. Hutton, M.D. Second Edition. Pp. x., 125. London: William Heinemann Ltd. 1929. Price 5s.—This small book sets out to put before men and women about to be, or already married, the facts of sex life, not in the prurient language of a certain author, but in normal medical and lay terms, and this it accomplishes admirably. Sex matters are " dealt with during all phases of married life," combined with easily understandable physiology and anatomy. In this, the second edition, fresh chapters have been added, including one on menstruation and the menopause. This chapter is especially necessary, and has added greatly to the value of the book. It can be recommended, without fear, to any woman seeking advice in matters of sex or personal hygiene.

**Burdett's Hospitals and Charities.** Pp. xxviii., 1,015. London: Nursing Mirror Ltd. 1930. Price 21s.—This invaluable reference work has reached its fortieth annual issue, and as time goes on its usefulness increases. It is known all the world over as the most reliable guide to all our hospitals, dispensaries and benevolent institutions. But the column for 1930 contains a review of current topics and problems connected with voluntary hospitals contributed by Captain J. E. Stone, which is of particular interest. The Local Government Act of 1929 aims at placing a complete hospital service within reach of everybody by means of an agreed policy between the voluntary hospitals and the local authorities. Captain Stone's article deals with this subject in an exhaustive manner, and contains besides useful details as to the various plans adopted by the hospitals in such questions as paying beds, contributory schemes, radium and cancer treatment. All who take any interest in the changes in hospital affairs which appear imminent will find Burdett's *Hospitals and Charities* the book of the hour.

